# The response to neoadjuvant chemoradiotherapy with 5-fluorouracil in locally advanced rectal cancer patients: a predictive proteomic signature

**DOI:** 10.1186/s12014-018-9192-2

**Published:** 2018-04-13

**Authors:** Anaïs Chauvin, Chang-Shu Wang, Sameh Geha, Perrine Garde-Granger, Alex-Ane Mathieu, Vincent Lacasse, François-Michel Boisvert

**Affiliations:** 1 Department of Anatomy and Cell Biology, Faculty of Medicine and Health Sciences, Université de Sherbrooke, 3201 Jean-Mignault, Sherbrooke, QC J1E 4K8 Canada; 2 Department of Nuclear Medicine and Radiobiology, Faculty of Medicine and Health Sciences, Université de Sherbrooke, Sherbrooke, QC Canada; 3 Department of Pathology, Faculty of Medicine and Health Sciences, Université de Sherbrooke, Sherbrooke, QC Canada

**Keywords:** Rectal cancer, Resistance to neoadjuvant radio-chemotherapy, 5-Fluorouracil, Proteomics, Mass spectrometry, Predictive biomarkers

## Abstract

**Background:**

Colorectal cancer is the third most common and the fourth most lethal cancer in the world. In the majority of cases, patients are diagnosed at an advanced stage or even metastatic, thus explaining the high mortality. The standard treatment for patients with locally advanced non-metastatic rectal cancer is neoadjuvant radio-chemotherapy (NRCT) with 5-fluorouracil (5-FU) followed by surgery, but the resistance rate to this treatment remains high with approximately 30% of non-responders. The lack of evidence available in clinical practice to predict NRCT resistance to 5-FU and to guide clinical practice therefore encourages the search for biomarkers of this resistance.

**Methods:**

From twenty-three formalin-fixed paraffin-embedded (FFPE) biopsies performed before NRCT with 5-FU of locally advanced non-metastatic rectal cancer patients, we extracted and analysed the tumor proteome of these patients. From clinical data, we were able to classify the twenty-three patients in our cohort into three treatment response groups: non-responders (NR), partial responders (PR) and total responders (TR), and to compare the proteomes of these different groups.

**Results:**

We have highlighted 384 differentially abundant proteins between NR and PR, 248 between NR and TR and 417 between PR and TR. Among these proteins, we have identified many differentially abundant proteins identified as having a role in cancer (IFIT1, FASTKD2, PIP4K2B, ARID1B, SLC25A33: overexpressed in TR; CALD1, CPA3, B3GALT5, CD177, RIPK1: overexpressed in NR). We have also identified that DPYD, the main degradation enzyme of 5-FU, was overexpressed in NR, as well as several ribosomal and mitochondrial proteins also overexpressed in NR. Data are available via ProteomeXchange with identifier PXD008440.

**Conclusions:**

From these retrospective study, we implemented a protein extraction protocol from FFPE biopsy to highlight protein differences between different response groups to RCTN with 5-FU in patients with locally advanced non-metastatic rectal cancer. These results will pave the way for a larger cohort for better sensitivity and specificity of the signature to guide decisions in the choice of treatment.

**Electronic supplementary material:**

The online version of this article (10.1186/s12014-018-9192-2) contains supplementary material, which is available to authorized users.

## Background

Colorectal cancer (CRC) is the third most common cancer (9.7%) in the world behind lung (13%) and breast (12%) cancers and the fourth most lethal (8.5%) behind lung (19%), liver (9.1%) and stomach (8.8%) cancers [1]. This high mortality can be explained by a late detection of the cancer, so patients are often diagnosed with locally advanced tumor completely invading the wall of the rectum (T3) or peripheral tissues or organs (T4), and often regional lymph node metastases are found (N1 or N2). In fact, the 5-year survival is estimated at 93, 78, 66 and 8% for stages I, II, III and IV respectively [2]. In North America, the standard treatment of locally advanced rectum cancer is neoadjuvant radiotherapy with concomitant chemotherapy with the intravenous 5-fluorouracil (5-FU) or its oral analogue Capecitabine (Xeloda^®^) followed by total mesorectal excision (TME) surgery [3]. This neoadjuvant treatment has been shown to reduce tumor infiltration and decrease the tumor charge (down-staging effect), which is important to increase the complete resection rate (R0, healthy circumferential margin) of surgery and thus improve loco-regional tumor control, disease-free survival (DFS) as well as quality of life by preserving sphincter, urinary and sexual functions [4–10].

While 70% of patients show an objective response (OR), including up to 15% with a complete remission [10–12], a subset of patients with the same tumor stage and having been treated with the same technique do not respond favourably, suggesting a resistant phenomenon. Moreover, considering the severe secondary effects observed such as radiodermatitis and proctitis, there is a strong need in clinic to have reliable biomarkers in order to predict the outcome of the treatment and to identify the sub-group of patients with resistant phenotype, permitting to avoid long, costly and ineffective procedures but with an adequate personalized treatment.

Many tests exist to detect CRC such as faecal occult blood test (FOBT), double contrast barium enema (DCBE) or colonoscopy, but biopsy remains one of the best ways to get a definitive diagnosis. A new blood test, the ColonSentry^®^ test, was recently developed and is based on the expression profile of seven genes *ANXA3*, *CLEC4D*, *LMNB1*, *PRRG4*, *TNFAIP6*, *VNN1* and *IL2RB* [13]. This rapid and non-invasive test represents the world’s first commercially available test for stratifying current risk for CRC. Of all these tests, it appears that the majority of them consist of screening tests but the clinical markers currently used for rectal cancer are not able to predict the individual response of the patient that would allow personalization of the treatment [14, 15]. Relevant biological factors to direct the patient to personalized treatment are lacking to guide the clinician’s decisions. The vast majority of predictive and prognostic biomarkers identified in rectal cancer generally involve genetic mutations such as the activating mutation in *KRAS* (G12D) which is a predictive biomarker of resistance to EGFR-directed therapy with the monoclonal antibodies cetuximab or panitumumab [16]. Furthermore, response to radiotherapy implicates a lot of factors in cell cycle arrest, DNA damage repair and apoptosis. So far, no reliable biomarker in clinic to predict the response pattern to neoadjuvant radio-chemotherapy, which is well associated with disease-free survival.

In this study, we focused on profiling the tumor protein signature of different groups of responders to NRCT with 5-FU: non-responders, partial and total responders (NR, PR and TR respectively) in patients with locally advanced rectal cancer. The purposes of this study are (1) to extract the protein content from rectal formalin-fixed paraffin-embedded (FFPE) biopsies and to study it by mass spectrometry, (2) to identify the differentially abundant proteins (3) to identify, from these proteins, the signaling pathways predominantly represented in each group and finally, (4) to discuss potential predictive biomarkers of the response to the NRCT with 5-FU.

## Methods

### Study design and clinical protocol

**Fig. 1:**
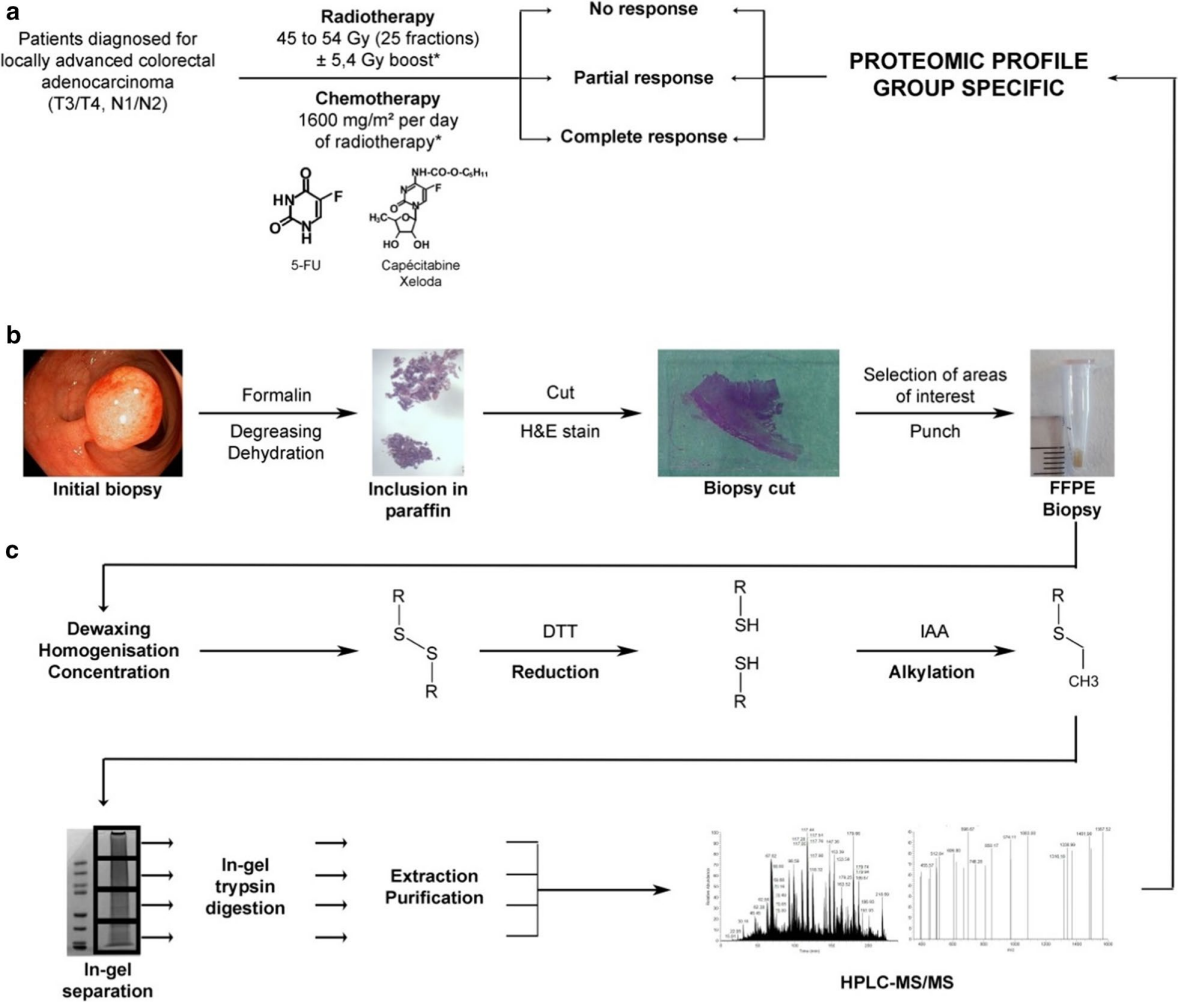
Overview of the workflow to obtain the group-specific proteomic profile. **a** Clinical selection and treatment. Patients with locally advanced non-metastatic colorectal cancer (CRC) are treated by radiotherapy (RT) with a concomitant chemotherapy (CT), in a neoadjuvant situation (preoperatively) (NRCT). (*) The doses indicated are the standard doses applied. The dose of RT is 45 to 54 Gray (Gy) in total, divided into 25 fractions (5 days per week for 5 weeks). The concomitant CT is 1600 mg/m^2^ per day of RT. It can be either intravenous (5-fluorouracil, 5-FU) or oral (Capecitabine or Xeloda^®^) (in the majority of cases, unless contraindications). After treatment, patients are divided in three groups according to their response to the treatment: no response (NR; absence of decreased stage/size of the tumor), partial response (PR; decreased stage/size of the tumor), and complete response (TR; elimination of the tumor). **b** From the initial sample to the FFPE biopsy: the physiopathological step. The fixation in formalin is immediately realized after the biopsy. The degreasing and dehydration are necessary for the inclusion in paraffin. The cutting and H&E staining steps allowed the pathologist to select the areas of interest and make a punch to obtain the FFPE biopsy (more details in Fig. 2). Punches are realized with TMA Master II (3DHISTECH). All these steps are realized in the laboratory of pathology. **c** From the FFPE biopsy to its proteomic profile: the experimental step. First steps (dewaxing, homogenisation and concentration) are necessary to realize a dithiothréitol reduction and an iodoacetamide alkylation on the samples. These last are in-gel separated and trypsin digested, and then the peptides are extracted and purified before their separation on HPLC–MS/MS. Analysis are realized with different software such as MaxQuant software package version 1.5.2.8 [18] and Perseus software package [19] and allowed to establish a proteomic profile specific for each group of responders. *5-FU* 5-fluorouracil, *CRC* colorectal cancer, *CT* chemotherapy, *FFPE* formalin-fixed paraffin-embedded, *Gy* Gray, *H&E* haematoxylin and eosin, *HPLC–MS/MS* high performance liquid chromatography separation coupled to mass spectrometry, *NR* non-responders, *PR* partial responders, *RT* radiotherapy, *TMA* tissue microarray, *TR* total responders

This is a retrospective study. The eligibility includes all patients diagnosed between September 2013 and September 2015 in the Centre Hospitalier Universitaire de Sherbrooke (Québec, Canada) with a pathologically confirmed locally advanced rectal adenocarcinoma (T3–T4, any N, M0/any T, N1–N2, M0), treated by standard neoadjuvant radiotherapy and chemotherapy followed by the surgery. All participants gave informed consent before enrolment in this study. The radiotherapy is composed by 45 Gy in 25 fractions, 5 days per week for 5 weeks, ± 5.4 Gy boost by 3D technique. The concomitant chemotherapy was performed by 1600 mg/m^2^ intravenous 5-FU or oral capecitabine (Fig. 1a). All secondary effects were reviewed and noted according to the Common Terminology Criteria for Adverse Events (CTCAE) version 4.0. The TNM stage before the treatment (iTNM) was used according to the routine clinical examination including magnetic resonance imaging (MRI) for all cases (Table 1) and the post-treatment TNM stage (ypTNM) was defined according to the report of pathology (Table 3). The cancer stage was finally defined according to the American Joint Committee on Cancer (AJCC). The tumor tissues of initial biopsy were used for the study. Patients were then classified into three distinct groups depending on their pathologic response to treatment evaluated with the AJCC tumor regression grading (TRG) system [17]. TRG0 corresponds to a complete regression (non-viable cancer cells), TRG1 corresponds to a near complete regression (single or small groups of tumor cells), TRG2 corresponds to a moderate regression (residual cancer outgrown by fibrosis) and TRG3 corresponds to a minimal/absent regression (minimal or no tumor cells killed). For the sake of clarity, we have associated TRG0 to the total responders (TR), TRG1 and TRG2 to the partial responders (PR) and TRG3 to the non-responders (NR). The study was approved by ethic committee of Cancer Research Center, CHUS (CRC-CHUS). Patients monitoring was performed on a regular basis to index recurrences.

**Table 1:** Baseline patient characteristics

Characteristics	All patients 23 (100)^a^	NR 7 (30)	PR 10 (43)	TR 6 (26)	*p* value^b^
Age (years)					
Median	58.6 ± 11.1	54.7 ± 14.3	59.7 ± 10.2	61.3 ± 8.6	
Range	31–75	31–72	47–75	48–71	
Sex					0.324
Male	19 (83)	7 (100)	7 (70)	5 (83)	
Female	4 (17)	0	3 (30)	1 (17)	
Tumor location from anal verge (cm)					0.961
3–5	10 (43)	3 (43)	5 (50)	2 (33)	
6–10	11 (48)	3 (43)	4 (40)	4 (67)	
> 10	2 (9)	1 (14)	1 (10)	0	
Tumor differentiation					0.884
Missing value	9 (39)	3 (43)	3 (30)	3 (50)	
Well	5 (22)	2 (29)	2 (20)	1 (17)	
Moderately	9 (39)	2 (29)	5 (50)	2 (33)	
Undifferentiated	0	0	0	0	
Pre-treatment stage					0.441
IIA	3 (13)	0	2 (20)	1 (17)	
IIIA	1 (4)	0	1 (10)	0	
IIIA–IIIB	2 (9)	0	0	2 (33)	
IIIB	4 (17)	1 (14)	2 (20)	1 (17)	
IIIB–IIIC	12 (52)	5 (71)	5 (50)	2 (33)	
IIIC	1 (4)	1 (14)	0	0	
iT					0.560
T2	3 (13)	0	1 (10)	2 (33)	
T3	18 (78)	6 (86)	8 (80)	4 (67)	
T4	2 (9)	1 (14)	1 (10)	0	

*iT* initial tumor, *NR* non-responders, *PR* partial responders, *TR* total responders

^a^First number: number of patients; number in parentheses: percentage of patients

^b^The *p* value is obtained by Fisher’s exact test and is considered significant when *p* value < 0.05

### Biopsy sample collection

**Fig. 2:**
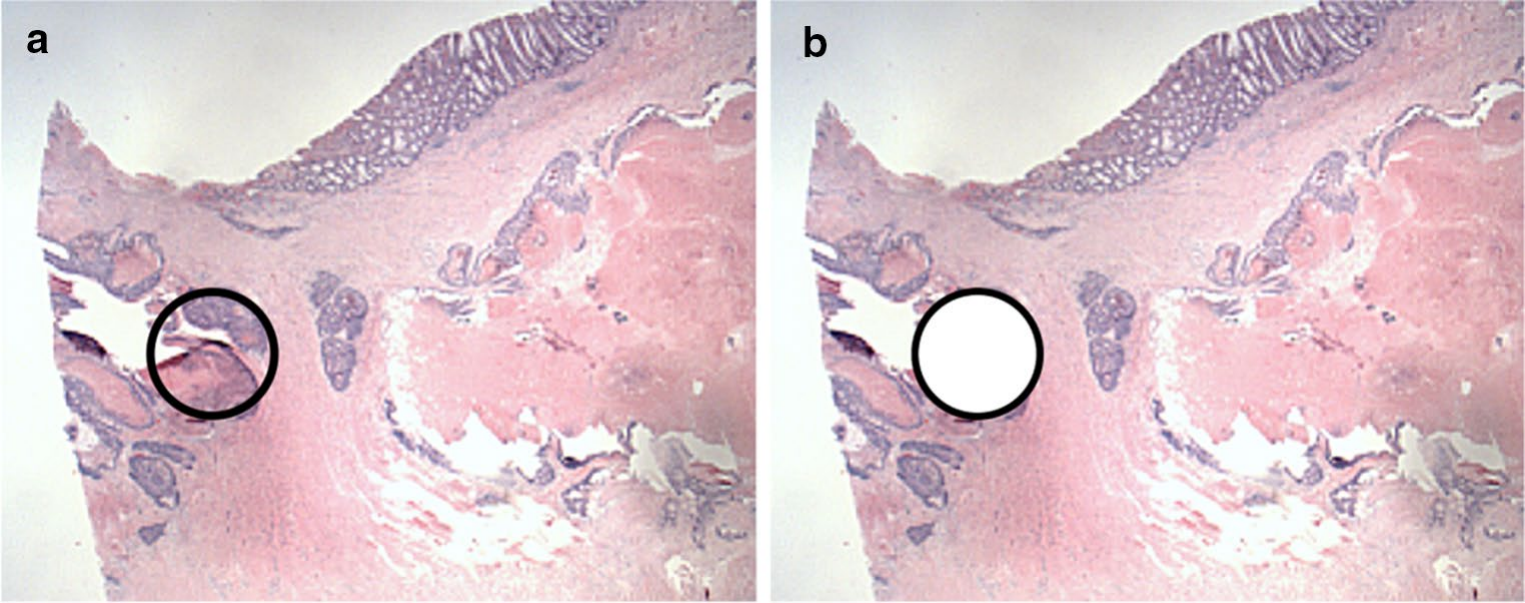
Cut obtained from a FFPE biopsy performed in a patient with locally advanced non-metastatic CRC (magnification ×1.5). **a** Selecting the area of interest containing the tumor cells by the pathologist. **b** Punch of the region of interest with the TMA Master II (3DHISTECH). *CRC* colorectal cancer, *FFPE* formalin-fixed paraffin-embedded, *TMA* tissue microarray

Biopsies were collected, rapidly fixed in formalin and sent to the pathology laboratory of the Centre Hospitalier Universitaire de Sherbrooke (Québec, Canada), according to the clinical pathological process. They have undergone washings (alcohols and solvents) in order to be degreased and dehydrated before their inclusion in paraffin. Cuts of approximately 3 μm in thickness were made on the paraffin blocks and analysed after staining with haematoxylin and eosin (H&E stain). The areas of interest were selected by the pathologist (Fig. 2a) and punches were made on the paraffin blocks (Fig. 2b) using a Tissue Microarray (TMA) Master II (3DHISTECH) (Fig. 1b).

### Proteomic study

#### Dewaxing and homogenisation of the FFPE biopsies

100% xylene was added (100 µl for 1 mg of biopsy) for 1 h under agitation. Xylene is replaced by 50% ethanol to dehydrate the biopsy for 1 h under agitation. After dehydration, FFPE biopsies were homogenised in MS grade water (300 µl for 1 mg of remaining biopsy) using a TissueRuptor (Qiagen). Biopsies were then incubated in 2.5% SDS, 50 mM Tris pH 7.5, 10 mM dithiothréitol at 98 °C for 1 h. After centrifugation at 13,000 rpm for 1 min, the supernatant was collected (Fig. 1c).

#### Concentration, reduction and alkylation

Solubilised biopsies were concentrated (250–50 µl) using Nanosep 3 K Omega (Pall Life Sciences) and treated with 1X Laemmli and 2 mM dithiothréitol at 98 °C for 5 min (reduction). 50 mM iodoacetamide was added for 30 min and incubated in the dark for alkylation (Fig. 1c).

#### In-gel separation and tryptic digestion

50 µg of concentrated biopsies were separated on NuPAGE gel 4–12% bis–Tris 1.5 mm × 10 well (Invitrogen). After migration, the gel was stained with Coomassie Blue (SimplyBlue SafeStain) for 1 h under agitation and then discoloured overnight under agitation. Each lane was divided into four gel slices. Each gel slice was cut into cubes (ca. 1 × 1 mm) and transferred in microcentrifuge tube. Then, they were washed with MS grade water for 15 min under agitation then with 50% acetonitrile for 15 min under agitation until gel slice shrunk and became opaque. The gel slices were then subjected to a wash cycle (20 mM ammonium bicarbonate for 15 min under agitation and 20 mM ammonium bicarbonate/100% acetonitrile (1:1, vol/vol) for 15 min under agitation) until the gel became clear. The samples were then dehydrated by washing with 100% acetonitrile for 5 min under agitation until the precipitate became white and solid. Acetonitrile was removed and dehydrated samples were digested in 12.5 ng/ml Trypsin Gold (Promega) modified in 50 mM acetic acid and 20 mM ammonium bicarbonate, at 37 °C overnight under agitation (Fig. 1c).

#### Peptides extraction

After the tryptic digestion, the supernatant was collected in a new microcentrifuge tube. Digested samples were next incubated with 100% acetonitrile (volume equal to the digestion volume in the previous step) for 30 min at 37 °C under agitation. The supernatant was collected and digested samples underwent two incubations in 1% formic acid for 20 min at 37 °C under agitation for extracting peptides from gel. The samples were then dehydrated by washing with 100% acetonitrile for 10 min at 37 °C under agitation until the precipitate became white and solid.

#### Peptides desalting

Peptides were resuspended in 30 µl of 0.1% trifluoroacetic acid. With a C18 column Zip-Tip (Thermo Scientific) previously washed with 100% acetonitrile and 0.1% trifluoroacetic acid, peptides were loaded into the column by doing 10 up and down, washed with 0.1% trifluoroacetic acid and eluted with 30 µl of 50% acetonitrile/1% formic acid by doing up and down in a new microcentrifuge tube. Desalted peptides were then dried in a speedvac and resuspended in 1% formic acid (Fig. 1c).

#### High performance liquid chromatography separation, coupled to mass spectrometry (HPLC–MS/MS)

Trypsin-digested purified peptides were separated using a Dionex Ultimate 3000 nanoHPLC system. 2 µg of the sample in 1% formic acid (v:v) were loaded with a constant flow of 4 µL/min on an Acclaim PepMap100 C18 column (0.3 mm id × 5 mm). After trap enrichment, peptides were eluted in a PepMap C18 nanocolumn (75 µm × 50 cm) with a linear gradient of 5–35% solvent B over 240 min with a constant flow of 200 nL/min.

#### Mass spectrometry analysis

The HPLC system was coupled to an OrbiTrap QExactive mass spectrometer (Thermo Fisher Scientific Inc) via an EasySpray source. The spray voltage was set to 2.0 kV and the temperature of the column was set to 40 °C. Full scan MS survey spectra (m/z 350–1600) in profile mode were acquired in the Orbitrap with a resolution of 70,000 after accumulation of 1,000,000 ions. The ten most intense peptide ions from the preview scan in the Orbitrap were fragmented by collision induced dissociation (normalized collision energy 35% and resolution of 17,500) after the accumulation of 50,000 ions. Maximal filling times were 250 ms for the full scans and 60 ms for the MS/MS scans. Precursor ion charge state screening was enabled and all unassigned charge states as well as singly, 7 and 8 charged species were rejected. The dynamic exclusion list was restricted to a maximum of 500 entries with a maximum retention period of 40 s and a relative mass window of 10 ppm. The lock mass option was enabled for survey scans to improve mass accuracy.

#### Quantification and bioinformatics analysis

Data were processed, searched and quantified using the MaxQuant software package version 1.5.2.8 with the protein database from UniProtKB (Homo sapiens, 16/07/2013, 88,354 entries) [18]. The following settings were used for the MaxQuant analysis: 2 miscleavages allowed; fixed modification was carbamidomethylation on cysteine; enzyme was trypsin (K/R not before P); variable modifications included in the analysis were methionine oxidation and protein N-terminal acetylation; mass tolerance of 7 ppm for precursor ions and 20 ppm for fragment ions. We performed quantification based on protein intensities. First, we calculated the sum of the protein intensities obtained for each biopsy. We then divided these protein intensities sums by the lowest protein intensity sum of the cohort to obtain a ratio. Each protein intensity for each biopsy was then normalized to this ratio so that the sums of the protein intensities for each biopsy were equal. Finally, the analysis of mass spectrometry data were next carried out with the Perseus software package [19] and subjected to a normalization (z-score) and an ANOVA statistical test (comparison of the three groups) or Student’s *T* test (comparison of two groups), both corrected with a *p* value of 0.05. The mass spectrometry proteomics data have been deposited to the ProteomeXchange Consortium via the PRIDE [20, 21] partner repository with the dataset identifier PXD008440.

#### Statistical analysis

The different groups of patients were first compared according to clinicopathological parameters. Due to a small number of patients per group, Fisher’s exact test was done with the R software [22]. When the *p* value was less than 0.05, the result was considered to have statistical significance.

## Results

### Baseline population characteristics

As the first step of study for the purpose of technique, 23 participants were enrolled and studied. Of the 23 patients, 19 were men (83%) and 4 were women (17%). The median age was 58.6 ± 11.1 years (31–75). Ten patients (43%) had low rectal cancer (within 5 cm from the anal verge), 11 patients (48%) had middle rectal cancer (between 5 and 10 cm from the anal verge) and 2 patients (9%) had proximal cancer (over of 10 cm from the anal verge) (Table 1). Tumor differentiation degrees defining by imaging were 5 well differentiated (22%), 9 moderately differentiated (39%) and 9 not specified (39%). TNM stages before NRCT (iTNM) were 3 stage IIA (13%), 1 stage IIIA (4%), 2 stage IIIA-IIIB (9%), 4 stage IIIB (17%), 12 stage IIIB-IIIC (52%) and 1 stage IIIC (4%) (Table 1).

### Surgical treatment and complications

Neoadjuvant radiotherapy was delivered to the pelvis with a dose of 45 Gy followed or not by a boost of 5.4 Gy for 21 patients (91%) and 1 patient (4%) respectively or with a dose of 50.5 (4%). Neoadjuvant chemotherapy was concurrently performed on all patients with Xeloda^®^ (oral) or 5-fluorouracil (intravenous) treatment (96 and 4% respectively). After NRCT, 16 patients (70%) had suffered from radiodermatitis (severity degrees are shown in Table 2). 12% of patients had suffered from proctitis and no cases of colitis or mucositis had been listed. On average, surgery delay after the beginning of treatment was 8.6 ± 2.8 weeks (1.4–13.1). Among the 23 patients, 3 patients refused the surgery. In these cases, the response is measured by MRI imaging after the treatment. The others 20 patients had a low anterior surgery (85%), or abdomino-perineal surgery (8%) even an endo-anal surgery (4%) (Table 2).

**Table 2:** Surgical treatment and complications

Characteristics	All patients 23 (100%)^a^	NR 7 (30%)	PR 10 (43%)	TR 6 (26%)	*p* value^b^
Radiotherapy					0.822
45 Gy	1 (4)	0	1 (10)	0	
45 Gy + 5.4 Gy	21 (91)	6 (86)	9 (90)	6 (100)	
50.5 Gy	1 (4)	1 (14)	0	0	
Chemotherapy					3.2e^−05^
Xeloda®	22 (96)	7 (100)	9 (90)	6 (100)	
5-FU	1 (4)	0	1 (10)	0	
Radiodermatitis					0.962
−	7 (30)	2 (29)	3 (30)	2 (33)	
Non-severe	13 (57)	4 (57)	5 (50)	4 (67)	
Severe	3 (13)	1 (14)	2 (20)	0	
Proctitis					0.211
+	11 (48)	2 (29)	7 (70)	2 (33)	
−	12 (52)	5 (71)	3 (30)	4 (67)	
Colitis					1
+	0	0	0	0	
−	23 (100)	7 (100)	10 (100)	6 (100)	
Mucositis					1
+	0	0	0	0	
−	23 (100)	7 (100)	10 (100)	6 (100)	
Surgical resection type					0.004
Low anterior	17 (74)	7 (100)	8 (80)	2 (33)	
Abdomino-perineal	2 (9)	0	2 (20)	0	
Endo-anal	1 (4)	0	0	1 (17)	
Refused	3 (13)	0	0	3 (50)	
Surgery delay					
Median	8.6 ± 2.8	9.3 ± 2.7	8.3 ± 3.1	8.1 ± 2.6	
Range	1.4–13.1	5.3–13.1	1.4–12.9	6.0–11.0	

*5-FU* 5-fluorouracil, *Gy* Gray, *NR* non-responders, *PR* partial responders, *TR* total responders

^a^First number: number of patients; number in parentheses: percentage of patients

^b^The *p* value is obtained by Fisher’s exact test and is considered significant when *p* value < 0.05

### Neoadjuvant chemoradiotherapy post-treatment results

After NRCT and surgery, the response to NRCT has been reported by pathological analysis for all patients operated and noted as standard pathological TNM classification (ypTNM) (Table 3). According to the definition of response and down-staging effects, all patients operated have been attributed in three distinct groups: NR, PR and TR. Among the 23 patients, there were 7 NR (30%), 10 PR (43%) and 6 TR (26%). Perineural and lymphovascular invasion (PNI and LVI) are searched on the surgical piece: 2 and 8 cases of PNI and LVI are listed respectively.

**Table 3:** Post-treatment patient characteristics

Characteristics	All patients 23 (100%)^a^	NR 7 (30%)	PR 10 (43%)	TR 6 (26%)	*p* value^b^
Tumor diff. after treatment					0.064
No surgery	3 (13)	0	0	3 (50)	
Missing value	5 (22)	2 (29)	1 (10)	2 (33)	
Well	5 (22)	1 (14)	4 (40)	0	
Moderately	9 (39)	3 (43)	5 (50)	1 (17)	
Undifferentiated	1 (4)	1 (14)	0	0	
Post-treatment stage					3.3e^−08^
0	6 (26)	0	0	6 (100)	
I	7 (30)	0	7 (70)	0	
IIA	3 (13)	2 (29)	1 (10)	0	
IIIA	2 (9)	0	2 (20)	0	
IIIB	2 (9)	2 (29)	0	0	
IIIB–IIIC	3 (13)	3 (43)	0	0	
ypT					4.6e^−08^
T0	6 (26)	0	0	6 (100)	
T1	3 (13)	0	3 (30)	0	
T2	6 (26)	0	6 (60)	0	
T3	7 (30)	6 (86)	1 (10)	0	
T4	1 (4)	1 (14)	0	0	
PNI					1
+	2 (9)	1 (14)	1 (10)	0	
−	21 (91)	6 (86)	9 (90)	6 (100)	
LVI					0.002
+	8 (35)	6 (86)	2 (20)	0	
−	15 (65)	1 (14)	8 (80)	6 (100)	

*LVI* lymphovascular invasion, *NR* non-responders, *PNI* perineural invasion, *PR* partial responders, *TR* total responders, *ypT* post-treatment tumor

^a^First number: number of patients; number in parentheses: percentage of patients

^b^The *p* value is obtained by Fisher’s exact test and is considered significant when *p* value < 0.05

### Identification of differentially abundant proteins between groups

**Fig. 3:**
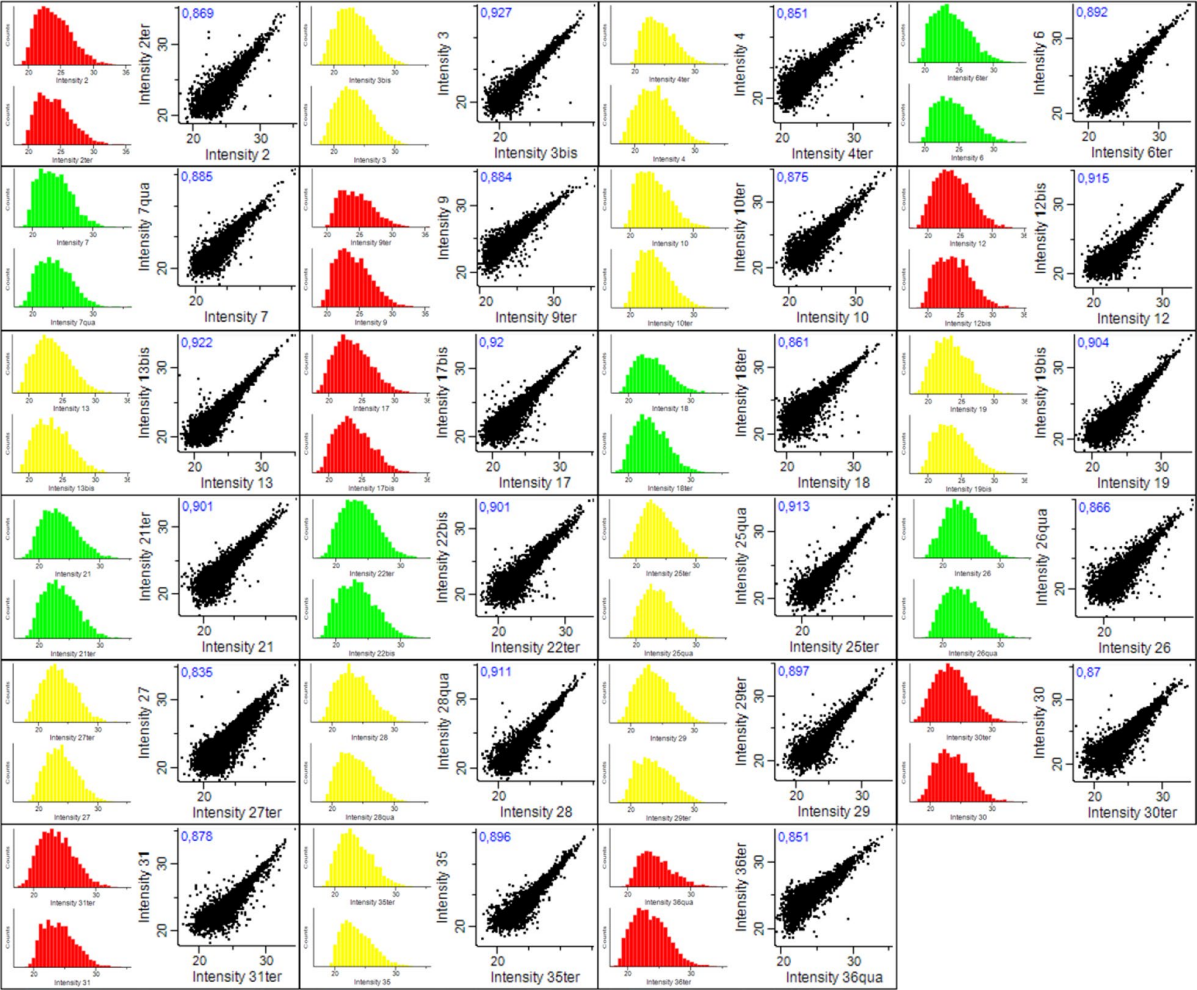
Distribution of protein intensities and multi-scatter plot for each biopsy. For each biopsy, on the left, histograms representing the distribution of protein intensities obtained (n = 1 and n = 2). The colors identify treatment response groups: total responders (TR) in green, partial responders (PR) in yellow and non-responders (NR) in red. For each biopsy, on the right, multi-scatter plot comparing the intensities obtained for n = 1 and n = 2. The Pearson correlation coefficient *r* is indicated in blue for each multi-scatter plot. *NR* non-responders, *PR* partial responders, *TR* total responders

We were able to identify 5329 proteins among the 23 patients of our study with on average more than 4000 proteins per sample. Using Perseus software package [19], we had first checked distribution of protein intensities for each duplicate biopsy and Pearson correlation coefficient *r* for each duplicate biopsy to ensure low variability within the same biopsy (n = 1 and n = 2) (Fig. 3).

**Fig. 4:**
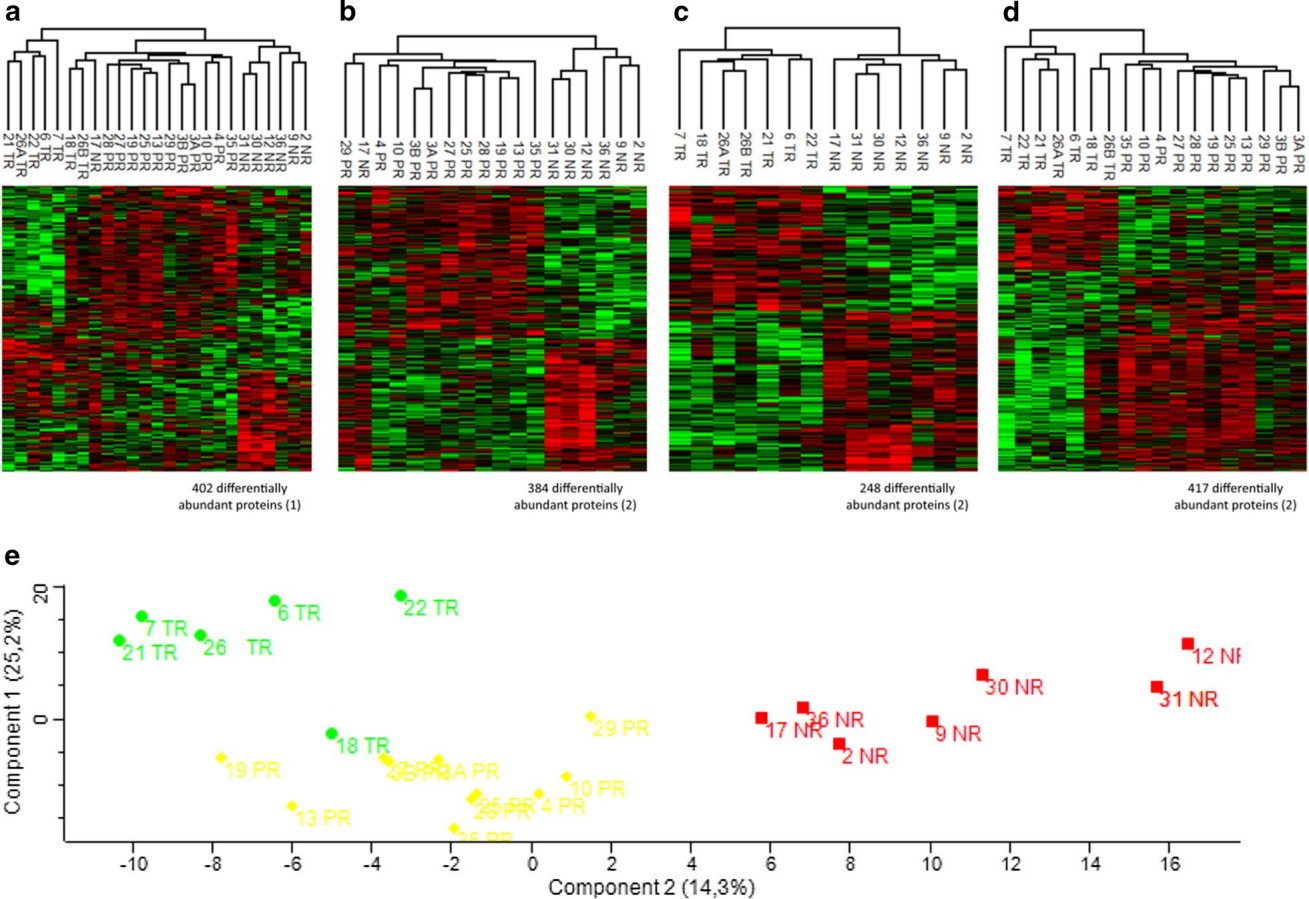
Heatmaps of differentially abundant proteins between groups of response and PCA plot showing the separation of these three groups. **a** Comparison between NR, PR and TR groups (*NR* non-responders, *PR* partial responders, *TR* total responders) resulting in 402 differentially abundant proteins. **b** Comparison between NR and PR groups resulting in 384 differentially abundant proteins (180 over-represented in NR and 204 in PR). **c** Comparison between NR and TR groups resulting in 248 differentially abundant proteins (139 over-represented in NR and 109 in TR). **d** Comparison between PR and TR groups resulting in 417 differentially abundant proteins (294 over-represented in PR and 123 in TR). Statistical tests used for these analyses are ANOVA test (1) and Student’s *T* test (2) both corrected with a *p* value of 0.05. **e** Principal component analysis (PCA) showing the separation between the three groups of response: TR in green diamond, PR in yellow circle and NR in red square. *NR* non-responders, *PCA* principal component analysis, *PR* partial responders, *TR* total responders

From that, we were able to identify 402 differentially abundant proteins between the three groups of responders (Additional file 1: Table S1) (Fig. 4a), 384 between NR and PR (180 over-represented in NR, 204 in PR) (Additional file 2: Table S2) (Fig. 4b), 248 between NR and TR (139 over-represented in NR, 109 in TR) (Additional file 3: Table S3) (Fig. 4c) and 417 between PR and TR (294 over-represented in PR and 123 in TR) (Additional file 4: Table S4) (Fig. 4d). Thanks to the principal component analysis (PCA) realized with the Perseus software package [19] (Fig. 4e), we can observe the groupings of the three responder groups NR, PR and TR. We also performed our analyses by grouping our patients into only two groups: responders and NR, (1) NR versus PR + TR, or (2) NR + PR versus TR. In the first case (1), we found 282 differentially abundant proteins, 139 of which were over-represented in NR and 143 in responders (PR + TR) (Additional file 5: Table S5). In the second case (2), 368 proteins were differentially abundant, of which 93 were over-represented in TR and 275 in non-responders (NR + PR) (Additional file 6: Table S6) (Table 4).

**Table 4:** Numbers of differentially abundant proteins between each group of responders

	Total	NR	PR	TR
NR versus PR versus TR^a^	402			
NR versus PR^b^	384	180	204	
NR versus TR^b^	248	139		109
PR versus TR^b^	417		294	123
NR versus PR + TR^b^	282	139	143	
NR + PR versus TR^b^	368	93	275	

*NR* non-responders, *PR* partial responders, *TR* total responders

Differentially abundant proteins obtained after ANOVA statistical test^a^ or Student’s *T* test^b^, both corrected with a *p* value of 0.05

In terms of protein identification (Additional file 7: Table S7), among the proteins expressed in more than 75% of the TR (≥ 5 patients) and in less than 15% of the NR (≤ 1 patient), we identified the following proteins: PVR, IFIT1, F12, FASTKD2, BPGM, PIP4K2B, MCMBP, CHD1L, ENSA, PVRL1, TRMT5, CNOT10, SLC25A33, FTO, AMDHD2, PHPT1, SLC5A6, LSM12, TSPAN6, CBX1, NOSIP, TSSC4, ARID1B, ALDH3A1, AAMDC, GTF2E1, SNX7 and STX10. In contrast, among the proteins present in more than 75% of the NR (≥ 5 patients) and in less than 15% of the TR (≤ 1 patient), we identified the following proteins (numbers in parentheses representing the relative abundance increase factor of NR over TR for each protein): CALD1 (4.4), CPA3 (9.4), B3GALT5 (55.9), CNNM4 (51.3), MTIF3 (4.1) and CD177 (2). By grouping our patients in two groups, for the first case (1), among the proteins expressed in more than 75% of the responders (PR + TR; ≥ 14 patients) and in less than 15% of the NR (≤ 1 patient), we identified FASTKD2, SLC25A33, FTO and AMDHD2 (proteins already mentioned above). In contrast, among the proteins present in less than 15% of the responders (PR + TR; ≤ 2 patients) and in more than 75% of the NR (≥ 5 patients), we only identified RIPK1 (3.7). For the second case (2), among the proteins expressed in more than 75% of the non-responders (NR + PR; ≥ 14 patients) and in less than 15% of the TR (≤ 1 patient), we only identified CALD1 (already mentioned above). In contrast, among the proteins present in less than 15% of the non-responders (NR + PR; ≤ 2 patients) and in more than 75% of the TR (≥ 5 patients), we identified IFIT1, ABLIM1, CNOT10 (proteins already mentioned above) and ERBB2.

**Fig. 5:**
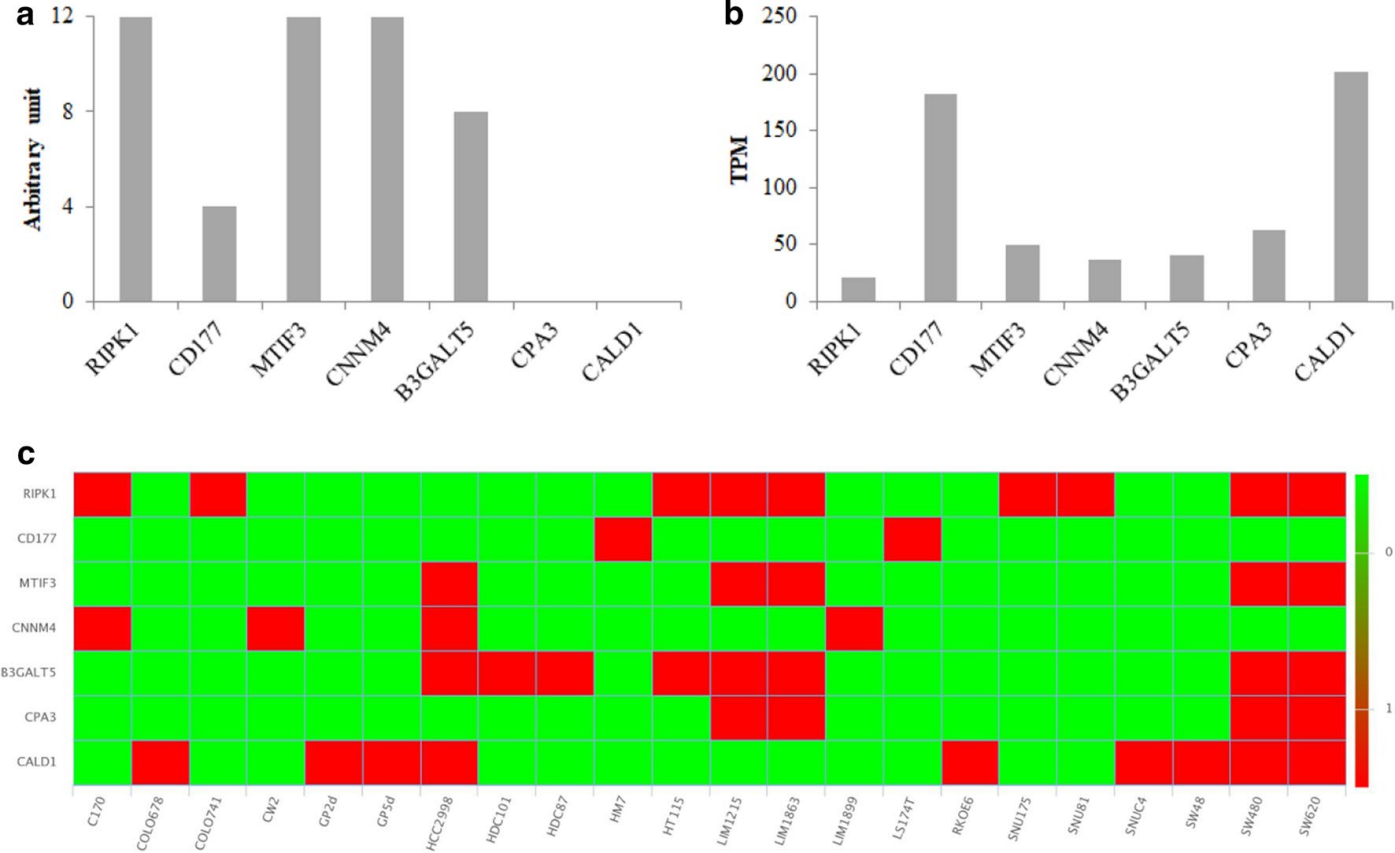
Protein and gene expression profile in normal rectal tissue and heat map for CALD1, CPA3, B3GALT5, CNNM4, MTIF3, CD177 and RIPK1 gene sequence variants in 22 colorectal cancer cell lines. **a** Protein expression profile in rectal tissue. The values are arbitrary units (0: undetected protein, 4: low expression, 8: medium expression, 12: high expression). These data were obtained from Human Protein Atlas available from http://www.proteinatlas.org. **b** Gene expression profile in rectal tissue. RNA-seq tissue data is reported as mean TPM (transcripts per million). These data were obtained from Human Protein Atlas available from http://www.proteinatlas.org. **c** Heat map of gene sequence variants in 22 colorectal cancer cell lines (in green: no sequence variants reported in coding region; in red: sequence variants reported in coding region). These data were obtained from http://colonatlas.org/index.html [23]

From Human Protein Atlas available from http://www.proteinatlas.org, we analysed the expression of the seven most abundant proteins in non-responders patients: CALD1, CPA3, B3GALT5, CNNM4, MTIF3, CD177 and RIPK1. These seven candidates are expressed in the normal rectal tissue at the protein level (Fig. 5a) and at the gene level (Fig. 5b). For CPA3 and CALD1, protein expression was not detected in the rectum but is expressed in the small bowel and colon respectively. In addition, there is a strong expression of their mRNAs in the rectal tissue. From Colorectal Cancer Atlas available from http://colonatlas.org/index.html [23], we were able to verify the expression of these seven proteins in 22 colorectal cancer cell lines as well as the presence of gene sequence variants (Fig. 5c). Many mutations have been identified for these genes but none are listed for involvement in deregulated signaling pathways in colorectal cancer.

We also observed the proteins involved in the metabolism of 5-FU. In the differentially abundant proteins, we find in particular the protein DPYD (dihydropyrimidine dehydrogenase), the enzyme involved in the hepatic catabolism of 5-FU. This protein is found to be more abundant in NR compared to TR (*P* = 3.18 × 10^−2^), in PR compared to TR (*P* = 2.63 × 10^−2^) and in NR + PR compared to TR (*P* = 1.87 × 10^−2^). We also find the protein TYMP (thymidine phosphorylase), the enzyme involved in the transformation of 5-FU into FdUMP (fluorodeoxyuridine monophosphate), which is the TS inhibitor (thymidylate synthase), the main target of 5-FU. This enzyme is found to be more abundant in PR compared to NR (*P* = 3.98 × 10^−5^), in PR compared to TR (*P* = 6.81 × 10^−3^) and in PR + TR compared to NR (*P* = 2.99 × 10^−3^).

**Fig. 6:**
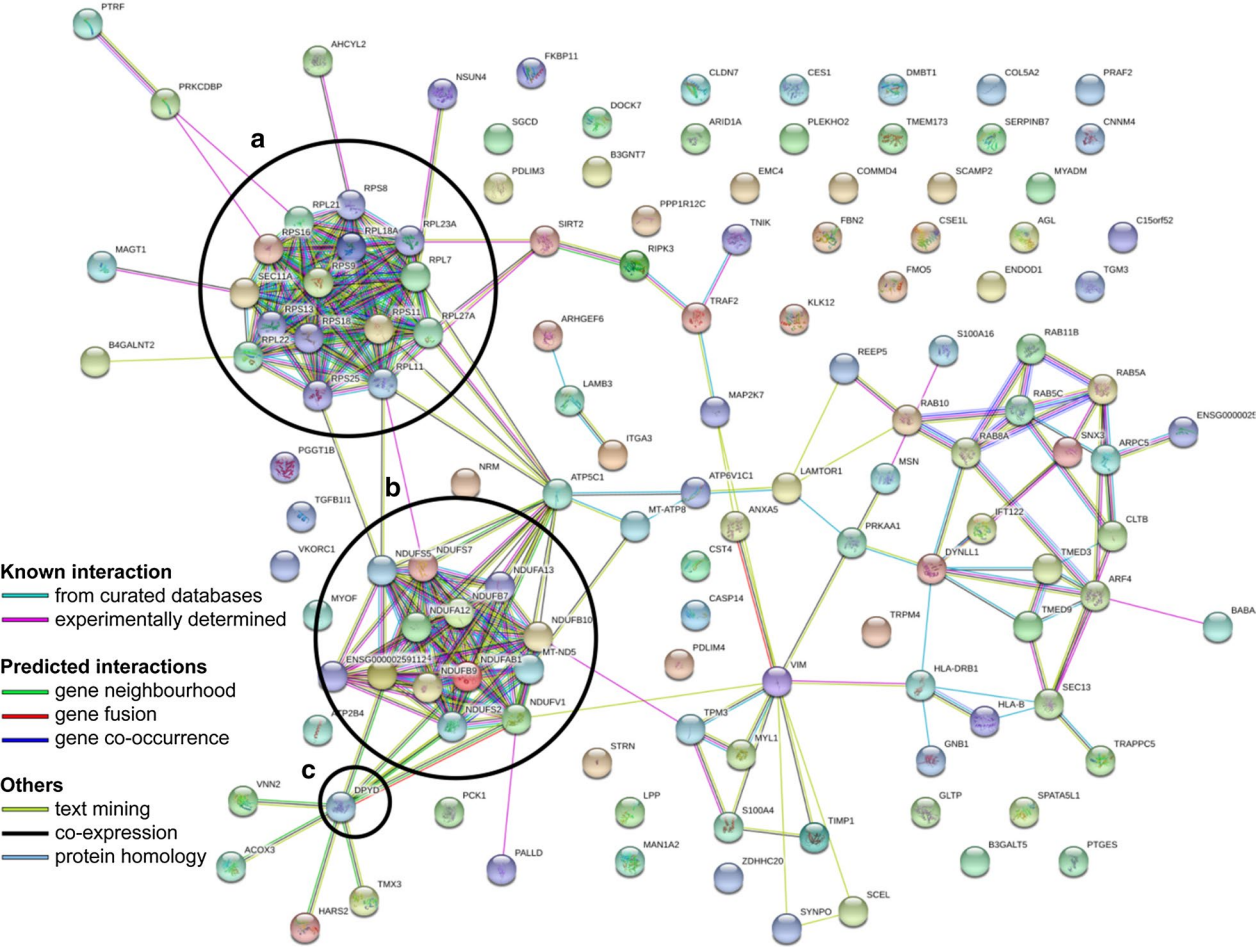
Representation by STRING software of differentially abundant proteins overexpressed in NR compared to TR (https://string-db.org/). The nature of the interactions is shown at the bottom left of the figure and lists the known interactions (from curated database, experimentally determined), the predicted interactions (gene neighbourhood, gene fusion, gene co-occurrence) and the others (text mining, co-expression, protein homology). Overexpressed proteins were found in NR versus TR with **a** ribosomal proteins (RPS and RPL), **b** NDUF mitochondrial respiratory chain complex I proteins and **c** DPYD, the enzyme responsible for 5-FU hepatic catabolism. *5-FU* 5-fluorouracil, *DPYD* dihydropyrimidine dehydrogenase, *NR* non-responders, *RPL* 40S ribosomal protein, small subunit, *RPS* 60S ribosomal protein, large subunit, *TR* total responders

From these differentially abundant proteins identified above, we looked for the biological processes predominantly represented in each group of responders for each comparison (Additional files 1–6: Tables S1–S6). These analyses were carried out using the DAVID (Database for Annotation, Visualization and Integrated Discovery) v6.8 software (https://david.ncifcrf.gov/) [24, 25]. Among these biological processes, there is a strong representation of ribosomal proteins (RP; RPS and RPL) and mitochondrial proteins (NDUF proteins, included in complexe I of the respiratory chain) in NR compared to the other groups as we can see in Fig. 6 obtained with the STRING software (https://string-db.org/) [26].

## Discussion

Neoadjuvant radio-chemotherapy with 5-FU is the standard treatment protocol for patients with locally advanced non-metastatic rectal cancer. However, there is still a very high resistance rate to this treatment (30% on average) and the mechanism for that individual resistance is not clearly understood. Studies looking for biomarkers predictive of this resistance have not yet led to the use of these biomarkers in the clinical decisions. FFPE biopsies have long been the subject of proteomic studies since they allow inexpensive and indefinitely storage at room temperature. Indeed, it has been shown that their protein composition after 3 or 15 years of storage was unchanged [27] and that a 92% overlap in the protein composition between a FFPE and fresh-frozen biopsies was obtained [28]. This model has even been described as a “treasure” for retrospective studies [29]. From the literature, we have developed a protein extraction protocol from FFPE biopsies of patients with locally advanced non-metastatic rectal cancer (Fig. 1) which allowed us to extract a satisfactory quantity of proteins identified by mass spectrometry, with an average of 4000 proteins identified by samples (Additional file 7: Table S7). In addition, the results presented in Fig. 3 with the distributions of protein intensities and multi-scatter plots for each biopsy allowed us to ensure low variability within duplicate samples. Protein compositions were finally compared between different treatment response groups: NR, PR and TR.

Firstly, we were interested in the proteins mainly abundant in each of these groups. For TR, we identified thirty proteins expressed in more than 75% of individuals in this response group and in less than 15% of NR: PVR, IFIT1, F12, FASTKD2, BPGM, PIP4K2B, MCMBP, CHD1L, ENSA, PVRL1, TRMT5, CNOT10, SLC25A33, FTO, AMDHD2, PHPT1, SLC5A6, LSM12, TSPAN6, CBX1, NOSIP, TSSC4, ARID1B, ALDH3A1, AAMDC, GTF2E1, SNX7, STX10, ABLIM1 and ERBB2.

Many of them have been identified in the past for cancer. In particular, the case of IFIT1 (interferon-induced protein with tetratricopeptide repeats 1) can be discussed. A study reported that high levels of IFIT1 mRNA were associated with radiation resistance in breast cancer [30]. In parallel, another team studied the protein expression of IFIT1 and associated a high level of this protein with better local survival without relapse [31]. They also concluded that protein expression may be a better measure for IFIT1 as it has been shown that this protein inhibits translation and cell growth in a dose-dependent manner and can regulate its own translation [31, 32].

Concerning FASTKD2 (FAST kinase domain-containing protein 2, mitochondrial), it was shown to be regulated by NRIF3 (nuclear receptor-interacting factor 3) and the DIF-1 (interferon regulatory factor 2-binding protein 2) complex and this regulation modulated apoptosis in mammary and pancreatic cancer cells [33, 34]. Indeed, the absence of FASTKD2 repression seems to lead to apoptosis in these cells [34]. A lower proportion of FASTKD2 in non-responder individuals in our study may be responsible for resistance to treatment-induced apoptosis in these individuals.

We also identified PIP4K2B (phosphatidylinositol 5-phosphate 4-kinase type-2 beta), of which a low expression has been associated with low survival, high grade, and increased tumor size in breast cancer [35]. Knockdown experiments of this protein have indeed promoted the epithelium-mesenchyme transition (EMT) via activation of the TGF-β pathway and resulted in a decrease in the tumor suppressor protein E-cadherin (CDH1) [35].

ARID1B (AT-rich interactive domain-containing protein 1B) is part of an SWI/SNF complex with among others ARID1A, its mutually exclusive homologue. This complex allows the remodeling of chromatin in ATP-dependent manner and plays an important role in cell proliferation, differentiation, development and tumor suppression. ARID1A is the third most significantly mutated gene in human rectal cancer, with a frequency of 39% in MSI type cancers [36–38]. It has also been shown that a decreased ARID1A expression results in inhibition of 5-FU-induced apoptosis in colorectal cancer cell lines [39]. Moreover, when ARID1A is mutated, there is an increase in the probability of losing the expression of ARID1B [40]. In our cohort, ARID1B is predominantly abundant in patients responding to 5-FU neoadjuvant chemoradiotherapy, which would imply the potential role of ARID1B in 5-FU resistance.

Finally, SLC25A33 (solute carrier family 25 member 33) is a mitochondrial transporter for pyrimidines and is essential for the metabolism of mitochondrial DNA and RNAs [41]. Since 5-FU is an anti-pyrimidine analogue [42], it would be possible that SLC25A33 participates in the import of 5-FU into the mitochondria to carry out its cytotoxicity by incorporating itself into the mitochondrial DNA and RNAs [43]. The absence of SLC25A33 in non-responder patients in our cohort raises the potential role of this protein in participating in 5-FU resistance mechanisms.

At the opposite, for NR, we identified seven proteins expressed in more than 75% of individuals in this response group and in less than 15% of TR: CALD1 (×4.4), CPA3 (×9.4), B3GALT5 (×55.9), CNNM4 (×51.3), MTIF3 (×4.1), CD177 (×2) and RIPK1 (×3.7).

Concerning CALD1 (caldesmon), this protein seems to display contradictory roles in cancer. On one side, CALD1 has been identified as a potential repressor of invasion into colorectal cancer cells [44]. In contrast, CALD1 has been described as a key component of TGF-β-directed EMT by its overexpression [45]. Recently, it was concluded that elevated expression of CALD1 in stromal cells of CRC patients was predictive of robustly shorter disease-free intervals [46].

For CPA3 (carboxypeptidase A3), it has been identified in a molecular signature of eleven genes for extracapsular spread in oral squamous cell carcinoma. This signature establishes a poor prognosis of outcome in patients without lymph node metastases [47]. B3GALT5 (beta-1.3-galactosyltransferase 5) has been identified as a predictor of postoperative recurrence and survival in patients with hepatic carcinoma and its high expression has been associated with advanced stages and poor outcome [48]. CD177 antigen has also been identified as a poor prognostic factor in patients with non-small cell lung cancer [49]. For RIPK1 (receptor-interacting serine/threonine-protein kinase 1), this protein transduces inflammatory signals and cell death after binding of death receptors, activation of pathogen recognition receptors and DNA damage. The balance between its pro-death and pro-survival functions is very complex and still need to be discussed [50–52].

Another study substantially similar to ours has also focused on the identification of proteins predictive of the response to 5-FU neoadjuvant chemoradiotherapy in patients with rectal cancer [53]. Using a 2D-DIGE quantitative proteomic approach, they analyzed the proteome of frozen biopsies and showed several proteins that could be predictive of patient nonresponse: fibrinogen β chain, three isoforms of actin, Serpin B9, peroxiredoxin 4 and two isoforms of cathepsin D. Although our patients have followed the same therapeutic regimen, we do not find the same proteins. This can be explained in several ways. (1) We used FFPE biopsies while they used frozen biopsies and their 2D-DIGE separation technique is also different from ours. (2) For patient grouping, we used the AJCC system (TRG0–TRG3) while Repetto and collaborators used the Mandard system (TRG1–TRG5) [17]. Based on their grouping, they consider that TRG1 and TRG2 form the group of “good” response whereas if we rely on our grouping, we would have classified their TRG2 patients in the group of partial response and not in the total response. This shows the importance of specifying the starting material, the extraction technique and the patient classification system used.

Secondly, as indicated in the results, we also identified proteins involved in 5-FU metabolism in the differentially abundant proteins: DPYD and TYMP, found to be overexpressed in NR and responders, respectively. DPYD is the enzyme responsible for the hepatic elimination of 5-FU at approximately 80% [54]. It allows the transformation of natural uracil and thymine pyrimidine bases into the inactive metabolites 5-FUH2 and 5-FTH2 (dihydrouracil and dihydrothymine), respectively [55]. Patients with partial or total deficiency for this enzyme will be at high risk of toxicity to 5-FU treatment that may result in death [56]. It has already been shown that high levels of DPYD mRNA correlate with 5-FU resistance [57] which is consistent with our results from the protein point of view. Regarding TYMP, also called PD-ECGF (platelet-derived endothelial cell growth factor), its role in 5-FU resistance is more controversial as reported by Elamin et al. [58]. This protein stimulates metastasis, invasion, angiogenesis and cell death evasion on the one hand, and on the other hand catalyses the transformation reaction of 5-FU into FdUMP, a compound that will competitively inhibit TS, the main target of 5-FU [59]. It has already been shown that high levels of TYMP mRNA correlate with 5-FU resistance [60]. On the contrary, in our study cohort, it is the treatment-responsive patients group who have higher protein levels in TYMP compared to NR.

Thirdly, we were interested in the biological processes mainly represented in each group, thanks to the DAVID v6.8 software. What stands out most of our analyses are the ribosomal (RP) and mitochondrial (MP) proteins mostly represented in NRs. Among the RPs, it finds in particular the proteins composing the small (40S) and the large subunit (60S) of the ribosomes, RPS and RPL respectively. Among the MPs, the group most represented is that of NDUF proteins. These proteins encoded by nuclear DNA are part of the complex I (NADH dehydrogenase [ubiquinone]) of the mitochondrial respiratory chain. Interestingly, a study by Marin-Vicente et al. [61] on the effects of 5-FU on the proteome of colorectal cancer cell lines revealed that the predominantly down-regulated proteins after this treatment were MPs and RPs. They also showed that the effect on these proteins was not due to a general stress-induced cellular response since treatment with raltitrexed, an inhibitor of TS, did not produce the same effect [61]. They concluded that the early effects on RPs and MPs were probably due to the effects of 5-FU on pre-rRNA maturation and mitochondrial ribosome biogenesis and/or tRNAs, respectively [61]. In the presence of 5-FU, the pre-rRNAs do not transform into mature rRNAs. There is then an accumulation of pre-rRNAs that will be polyadenylated and degraded by the exosome. RPs will also accumulate in the nucleoplasm, interact with MDM2 and engage the 5-FU-mediated apoptotic response, and will be degraded. A higher representation of RPs in the NR patients’ tumor proteome in our cohort may be responsible for a lower response to 5-FU treatment. Regarding NDUF proteins, they are identified in a Zhang et al. [38] study on the proteogenomic characterization of colorectal cancer. This team has established signatures for the different proteomic subtypes established from many factors and NDUF proteins are found down-regulated within several of these signatures but not discussed [38]. A higher representation of NDUF proteins within the tumor proteome of NR individuals in our cohort may be implicated in a lower sensitivity to 5-FU treatment.

## Conclusions

In conclusion, our retrospective study aims to identify a predictive protein signature of the NRCT response to radiotherapy with 5-FU in patients with locally advanced non-metastatic rectal cancer from FFPE biopsies obtained in pre-treatment. Our results allowed us to validate our protein extraction protocol for the final realization of MS experiments. From the 23 patients in our cohort, we were able to establish three response groups, 6 NR, 10 PR and 7 TR, and compared the tumor proteomes. We have identified many differentially abundant proteins already identified as having a role in cancer, such as the earlier discussed proteins IFIT1, FASTKD2, PIP4K2B, ARID1B and SLC25A33 (more abundant in TR) or CALD1, CPA3, B3GALT5, CD177 and RIPK1 (more abundant in NR). We have also demonstrated proteins of the metabolism of 5-FU, DPYD and TYMP, and obtained results similar to those obtained in the literature in particular for DPYD, whose strong expression is associated with a poorer response to 5-FU. Finally, the detection of RPs and MPs seems to open the way for potential new mechanisms of resistance to 5-FU. By increasing the size of our cohort and moving to targeted proteomics experiments to detect and quantify the proteins of interest, we expect to define a protein signature to develop a test that could determine whether patients should be treated by RCTN with 5-FU.

## Additional files

**Additional file 1: Table S1.** Proteins differentially expressed between NR, PR and TR.

**Additional file 2: Table S2.** Proteins differentially expressed between NR and PR.

**Additional file 3: Table S3.** Proteins differentially expressed between NR and TR.

**Additional file 4: Table S4.** Proteins differentially expressed between PR and PR.

**Additional file 5: Table S5.** Proteins differentially expressed between non-responders (NR) and responders (PR+TR).

**Additional file 6: Table S6.** Proteins differentially expressed between non-responders (NR + PR) and total responders (TR).

**Additional file 7: Table S7.** Proteins identification (NR, PR and TR).

## Abbreviations

5-FU: 5-fluorouracil
ARID1B: AT-rich interactive domain-containing protein 1B
B3GALT5: beta-1.3-galactosyltransferase 5
CALD1: caldesmon
CDH1: E-cadherin
CPA3: carboxypeptidase A3
CRC: colorectal cancer
CT: chemotherapy
CTCAE: common terminology criteria for adverse events
DAVID: database for annotation, visualization and integrated discovery
DFS: disease-free survival
DIF-1: interferon regulatory factor 2-binding protein 2
DCBE: double contrast barium enema
DPYD: dihydropyrimidine dehydrogenase
EGFR: epidermal growth factor receptor
EMT: epithelium-mesenchyme transition
FASTKD2: FAST kinase domain-containing protein 2, mitochondrial
FdUMP: fluorodeoxyuridine monophosphate
FFPE: formalin-fixed paraffin-embedded
FOBT: faecal occult blood test
Gy: Gray
HPLC: high performance liquid chromatography
IFIT1: interferon-induced protein with tetratricopeptide repeats 1
iTNM: initial tumor, nodes, metastasis
LVI: lymphovascular invasion
MRI: magnetic resonance imaging
MS: mass spectrometry
NR: non-responders
NRCT: neoadjuvant radio-chemotherapy
NRIF3: nuclear receptor-interacting factor 3
OR: objective response
PCA: principal component analysis
PD-ECGF: platelet-derived endothelial cell growth factor (= TYMP)
PIP4K2B: phosphatidylinositol 5-phosphate 4-kinase type-2 beta
PNI: perineural invasion
PR: partial responders
RIPK1: receptor-interacting serine/threonine-protein kinase 1
RP: ribosomal proteins
RPL: 60S ribosomal protein, large subunit
RPS: 40S ribosomal protein, small subunit
RT: radiotherapy
SLC25A33: solute carrier family 25 member 33
TMA: tissue microarray
TME: total mesorectal excision
TNM: tumor, nodes, metastasis
TPM: transcripts per million
TR: total responders
TRG: tumor regression grading system
TS: thymidylate synthase
TYMP: thymidine phosphorylase (= PD-ECGF)
ypTNM: post-treatment tumor, nodes, metastasis
